# Late-acting dominant lethal genetic systems and mosquito control

**DOI:** 10.1186/1741-7007-5-11

**Published:** 2007-03-20

**Authors:** Hoang Kim Phuc, Morten H Andreasen, Rosemary S Burton, Céline Vass, Matthew J Epton, Gavin Pape, Guoliang Fu, Kirsty C Condon, Sarah Scaife, Christl A Donnelly, Paul G Coleman, Helen White-Cooper, Luke Alphey

**Affiliations:** 1 Department of Zoology, University of Oxford, South Parks Road, Oxford OX1 3PS, UK; 2 Oxitec Limited, 71 Milton Park, Oxford OX14 4RX, UK; 3 Department of Infectious Disease Epidemiology, Imperial College Faculty of Medicine, Norfolk Place, London, W2 1PG, UK; 4 Department of Infectious & Tropical Diseases, London School of Hygiene & Tropical Medicine, Keppel Street, London WC1E 7HT, UK

## Abstract

**Background:**

Reduction or elimination of vector populations will tend to reduce or eliminate transmission of vector-borne diseases. One potential method for environmentally-friendly, species-specific population control is the Sterile Insect Technique (SIT). SIT has not been widely used against insect disease vectors such as mosquitoes, in part because of various practical difficulties in rearing, sterilization and distribution. Additionally, vector populations with strong density-dependent effects will tend to be resistant to SIT-based control as the population-reducing effect of induced sterility will tend to be offset by reduced density-dependent mortality.

**Results:**

We investigated by mathematical modeling the effect of manipulating the stage of development at which death occurs (lethal phase) in an SIT program against a density-dependence-limited insect population. We found late-acting lethality to be considerably more effective than early-acting lethality. No such strains of a vector insect have been described, so as a proof-of-principle we constructed a strain of the principal vector of the dengue and yellow fever viruses, *Aedes *(*Stegomyia*) *aegypti*, with the necessary properties of dominant, repressible, highly penetrant, late-acting lethality.

**Conclusion:**

Conventional SIT induces early-acting (embryonic) lethality, but genetic methods potentially allow the lethal phase to be tailored to the program. For insects with strong density-dependence, we show that lethality after the density-dependent phase would be a considerable improvement over conventional methods. For density-dependent parameters estimated from field data for *Aedes aegypti*, the critical release ratio for population elimination is modeled to be 27% to 540% greater for early-acting rather than late-acting lethality. Our success in developing a mosquito strain with the key features that the modeling indicated were desirable demonstrates the feasibility of this approach for improved SIT for disease control.

## Background

Around the world, the medical and economic burden caused by vector-borne diseases continues to grow as current control measures fail to cope. There is an urgent need to identify new control strategies that will remain effective, even in the face of growing insecticide and drug resistance [1]. The Sterile Insect Technique (SIT) is a species-specific and environmentally benign method for insect population control that relies on the mass-rearing and release of sterile insects [2-4]. These released insects compete for mates with wild males; a wild female mating with a released sterile male has no or fewer progeny, so the population tends to decline. If sufficient sterile insects are released for a sufficient period, the target population will be controlled or even locally eradicated. SIT has been used successfully for over 50 years for area-wide control and/or elimination of several important agricultural pests and disease vectors, including the Mediterranean fruit fly [5], the screwworm fly [6,7] and the tsetse fly [8].

Though a number of trials were conducted in the 1970s, with some success, there are today no large-scale SIT programs in operation against any mosquito species [9,10]. The mosquito *Aedes aegypti *(also known as *Stegomyia aegypti*) is the key vector of the viruses that cause yellow fever and dengue fever. Dengue fever has seen a four-fold increase in incidence since 1970 and is a major public health problem threatening an estimated 2.5 billion people worldwide, with 50–100 million new infections per year[11,12]. *Ae. aegypti *is a robust mosquito species, suitable for mass-rearing. This species also appears to be reasonably homogeneous over large areas, without the problems of cryptic sub-species and barriers to mating and gene flow that have been found for some *Anopheles *mosquitoes [13,14]. For mosquitoes, male-only release is considered essential since sterile females will bite and so may transmit disease, whereas male mosquitoes do not bite. The early *Aedes *SIT trials showed that, even on a large scale, sex-separation based on pupal size can consistently give an essentially male-only population for release (< 1% female; as low as 0.1% female if larger males are also discarded [15]). A key difficulty for mosquito SIT is sterilization. Irradiation of pupae appears to damage the insects; irradiation as adults is less damaging but operationally far more difficult [16-19]. Some trials used sterilizing chemicals such as thiotepa [20-22], which proved effective for sterilization but led to trace contamination with this mutagenic chemical [23].

Another problem for mosquito SIT relates to the population biology of mosquitoes. Unlike agricultural pests, against which the major SIT programs are currently directed, mosquito populations may be regulated primarily by density-dependent effects, in which a highly fecund population is maintained at a stable level by resource limitation, e.g. availability of oviposition sites or nutrients for the larvae. Even a several-fold reduction in the average reproductive potential of females might therefore have no significant impact on the target population [24,25]. SIT programmes have generally been directed against agricultural pests which are not resource-limited, at least not limited by availability of larval food – which would imply that they have eaten the entire crop. While this may apply to a few agricultural pests under particular circumstances, such as locusts or gypsy moths, agricultural pests are more typically limited by seasonal effects and grower interventions rather than intraspecific competition for resources such as larval food.

We have previously proposed that insects engineered to carry a dominant lethal genetic system could be used to replace the need for radiation-sterilization in a SIT-like control program [26,27]. We named this approach RIDL (Release of Insects carrying a Dominant Lethal). Irradiation acts by inducing random dominant lethals; in RIDL this is replaced by an engineered dominant lethal. In both RIDL and SIT, some or all of the progeny of released individuals die as a consequence of inheriting one or more dominant lethal mutations, so the population tends to decline. For population control purposes, the only timing requirement for the lethal system is that death occurs before reproductive maturity. However, it is clearly desirable that death occurs before the point at which the insects cause harm. For mosquitoes, this is biting by adult females or, for disease transmission, female biting after a prior infected blood meal followed by pathogen incubation within the mosquito. Any lethal phase prior to adulthood is therefore acceptable. Radiation sterilization generates random dominant lethal mutations in the affected gametes, so the eggs laid by females inseminated by sterile males die early in embryogenesis and do not form larvae ("embryonic lethality"). This very early lethal phase clearly satisfies the condition "before the point at which the insect causes harm", but is not the only possibility. We here propose an alternative strategy, the use of a late-acting lethal, for example a pupal lethal, and show that it can be significantly more effective than conventional SIT at controlling populations limited by density-dependent effects, e.g. limited availability of larval nutrition.

## Results and Discussion

### Density-dependence

Density-dependent effects can stabilize a population at a given level, the "carrying capacity", so that, if perturbed in either direction, it will tend to return to that level. This is a common situation for wild populations, for example where individuals compete for limited resources such as food. It has previously been reported that, in some such cases, an SIT program with an insufficient release ratio could actually *increase *the stable adult population [24]; this prediction depends on assumptions that are not unreasonable for some mosquito populations. *Aedes aegypti *lays its eggs in small pools of relatively clean water, e.g. rainwater, with relatively little organic matter and therefore limited nutritional resources. *Aedes *pupae do not feed; competition for resources is predominantly in the larval phase. Sterilization with radiation or chemicals would typically result in progeny that die early in embryonic development, and so would not compete with their con-specifics for resources. This would lead to increased survival of the remaining larvae, an effect that would partly or completely offset the effect of killing some of the embryos [24,25]. In contrast, in a RIDL program using a strain with a late lethal phase, "doomed" heterozygotes would compete for resources as larvae and so tend to reduce the survival of their con-specifics. This could potentially help to offset the "rebound" effect expected for mosquito populations in the early stages of a conventional SIT program, reducing the required release ratio, and hence the cost and sustainability of the program. We investigated this potentially beneficial effect by mathematical modeling.

### Modeling early-acting and late-acting lethality for *Aedes aegypti*

**Figure 1:**
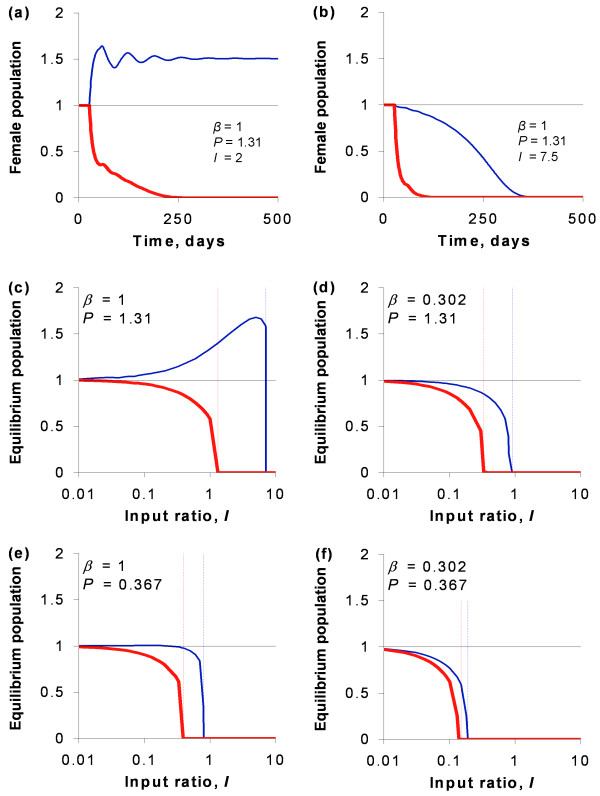
**Dynamics and equilibrium conditions of density-dependent-limited populations under RIDL/SIT control**. We compared the effectiveness of SIT (blue line) and a late-acting lethal RIDL strategy (thick red line) in a mathematical model of a continuous breeding *Ae. aegypti *population limited by density-dependent mortality (for details of the model see Methods). The population is assumed to start at equilibrium carrying capacity, and will therefore remain at the initial level if there is no intervention (black line). All releases are assumed to be of males only; the input release ratio, *I*, is defined relative to the initial wild male population; this rate of release of males then remains constant through time. In panels A and B, we plotted examples of the variation over time, from the start of control, of the number of females in the population relative to the initial number, for two different release ratios. The RIDL insects are assumed to be homozygous for a construct lethal to males and females ("non-sex-specific") *after *the density-dependent phase. For conventional SIT, mortality is assumed to be early (at embryogenesis), *before *any density-dependent mortality operates. With a low release ratio (A), SIT can actually increase the equilibrium size of the adult female population while RIDL can result in eradication. With a sufficiently high release ratio (B), conventional SIT can control the population, but the RIDL strategy is more effective. In panels C, D, E and F, we plot the equilibrium number of female mosquitoes in the population, relative to the initial numbers, following control with a given input ratio. The critical input ratios required to achieve eradication are shown as broken lines for the conventional SIT (blue) and RIDL systems (red). *β *represents the intensity of the density-dependence; *P *is the maximum per capita daily egg production rate corrected for density-independent egg to adult survival (see Methods). Parameter values for *β *and *P *(indicated in the panels) represent the best-estimate ranges calculated by Dye for a natural *Ae. aegypti *population [25]. In all cases, *T *= 27 days and *δ *= 0.12 per day; parameter values again taken from Dye [25].

We compared the effectiveness of an early-acting lethal, such as conventional SIT or an engineered lethal giving an early lethal phase, with that of a RIDL system inducing later-acting lethality. We used a mathematical model and parameters for density-dependence derived from field studies of *Aedes aegypti *[25]. We found that, for a population limited by density-dependent effects, delaying the time of death until after the density-dependent mortality phase has a strongly beneficial effect on program effectiveness (Fig. 1).

Under the assumptions of Fig. 1, use of a late-acting lethal is shown to be the more effective for any level of control effort, across the range of density-dependent and growth rate parameters estimated for a natural *Ae*. *aegypti *population [25]. Relative to an early-acting lethal, use of a late lethal not only lowers the critical input ratio required to achieve eradication (Fig. 1c–f), but also drives the population more rapidly to the new control-mediated equilibrium (Fig. 1a, b). These benefits are more pronounced where density-dependent effects are more pronounced, under which circumstances the threshold release ratios for population elimination are also higher (Fig. 1c). Importantly, use of a later lethal phase avoids any unintended increase in the mosquito equilibrium population relative to no control that may occur with an early-acting lethal under intense density-dependence (Fig. 1c, e). Even if the release rate is sufficient to avoid this over most of the program area, for an early-acting lethal, there is a risk that areas at or beyond the edge of the release area may incur this problem. Avoiding such potentially deleterious consequences of intervention, while reducing the effort and time required to achieve successful population suppression, are important potential benefits of such a system.

The effectiveness of a RIDL strategy based on the use of a late-acting lethal, relative to conventional SIT, is likely to be even greater than Fig. 1 illustrates because our model does not account for several advantages of RIDL, such as reduced production costs and the competitiveness advantage that transgenic males potentially have over irradiated males. Use of a repressible lethal has the additional advantage of a "fail-safe" protection against the consequences of accidental release of mass-reared insects: unlike conventional SIT, such insects require a dietary additive for survival and therefore cannot establish in the wild [28,29].

### A mosquito strain with conditional late-acting dominant lethality

**Figure 2:**
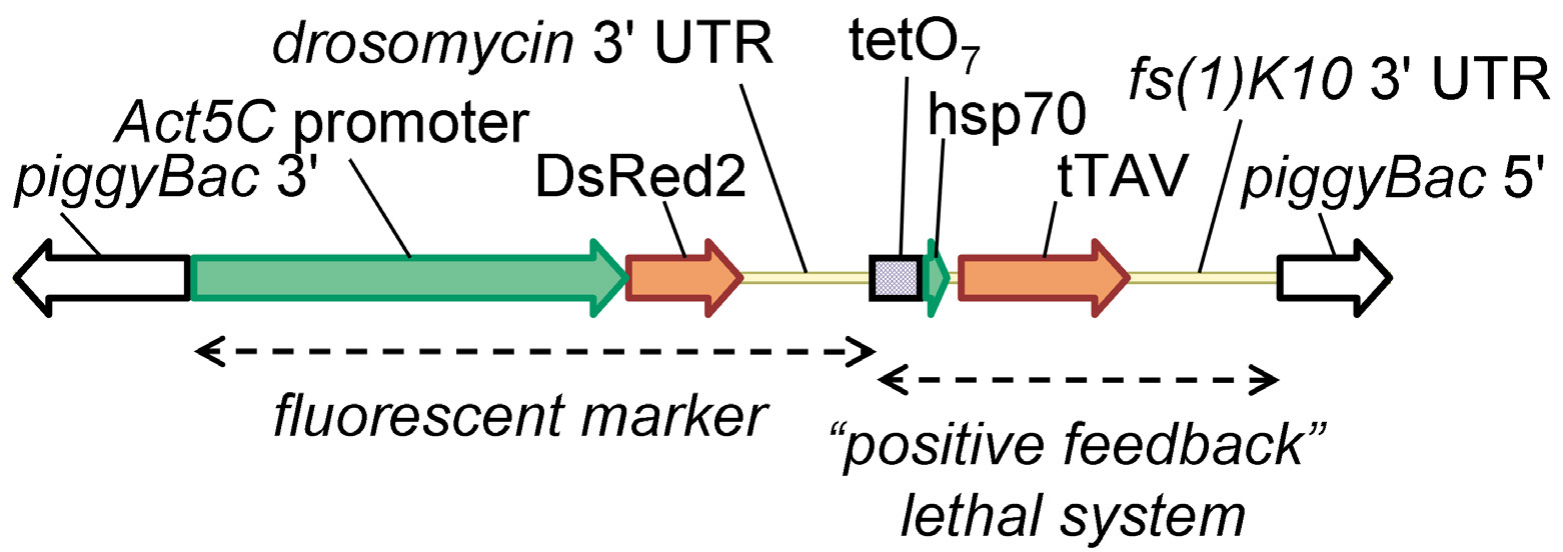
**The structure and function of transposon LA513**. LA513 is a non-autonomous *piggyBac*-based transposon of 8.4 kb. Transgenics are readily identified by red fluorescence due to expression of DsRed2. tTAV is a tetracycline-repressible transcriptional activator [28, 48]. Here, tTAV is under the control of its own binding site, tetO, a minimal promoter from *Drosophila *hsp70, and a 3' UTR sequence from *Drosophila fs(1)K10 *[49]. In the absence of tetracycline, tTAV binds to tetO and drives expression of more tTAV, in a positive feedback loop. In the presence of tetracycline, tTAV binds tetracycline; this tetracycline-bound form does not bind tetO and so does not lead to expression of more tTAV. Consequently, this construct gives very high levels of expression of tTAV in the absence of tetracycline, but only low, basal expression in the presence of tetracycline. High level expression of tTAV is toxic, possibly due to the interaction of the VP16 domain with key transcription factors, so this construct provides a tetracycline-repressible lethal system [28]. Construct LA882 is very similar to LA513; the principal difference is the use of the IE-2 promoter from the baculovirus *Op*NPV to drive expression of the DsRed2 marker, in place of *Act5C*.

Can a mosquito strain with the necessary properties – repressible dominant lethality acting at a larval or pupal stage – actually be constructed? We transformed *Ae. aegypti *with two molecular constructs, LA513 and LA882 (Fig. 2), predicted to induce tetracycline-repressible dominant lethality in both males and females. We had previously found constructs of similar design to give late-acting, primarily pupal, lethality in an agricultural pest insect, the Mediterranean Fruit Fly *Ceratitis capitata *[28]. We recovered four transgenic lines, LA513A, LA513B, LA882A and LA882B. All four appeared to be caused by insertions at single sites in the genome, as judged by the segregation pattern of the transgene over several generations of back-crossing to wild type (data not shown). Of these four lines, three gave highly penetrant (95–100%) tetracycline-repressible dominant lethality. Two of these, LA513B and LA882A, killed affected individuals as early larvae (L1–L3), which might give some degree of competition above that predicted for embryonic lethals, but is not ideal for this purpose. The third line, LA513A, killed affected individuals around the larval-pupal boundary, so we investigated this line further. This variation in phenotype between insertion lines is known as "position effect" and is a well-known consequence of the single-copy integration into random sites typical of insect transformation; we have observed similar effects with equivalent constructs in other species ([28] and data not shown).

We found no difference in the survival of LA513A/+ transgenics and their wild type siblings when reared in the presence of 30 μg/ml tetracycline, but in the absence of tetracycline only 3–4% of the transgenics survived from the first larval instar to adulthood, compared with 86–88% of wild type, a 95–97% reduction in survival relative to wild type (Table 1). The LA513A line therefore has a dominant lethal genetic system that is highly penetrant and appears to be fully repressible.

We determined the lethal phase of LA513A in the absence of tetracycline by crossing LA513A/+ heterozygotes to wild type and monitoring the survival of progeny carrying a single copy of the transgene (LA513A/+ heterozygotes), relative to their non-transgenic siblings (Table 1). This insertion was found by inverse PCR to be a precise integration at a TTAA target site (Suppl. Fig. 1B). The hatch rates of transgenic and wild type larvae were not significantly affected by the presence or absence of dietary tetracycline (binomial exact test, *p* = 0.248). We found no difference in survival to adulthood when the larvae were reared in the presence of 30 μg/ml tetracycline (χ^2 ^= 0.002, d.f. = 1, *p *> 0.1). In the absence of tetracycline, highly significant lethality was observed in transgenic individuals relative to wild type (χ^2 ^= 285, d.f. = 1, *p *< 0.001). Lethality of LA513/+ was predominantly at the larval/pupal boundary; most affected individuals that started to pupate failed to develop beyond a very early pupal stage. The LA513A line therefore carries a late-acting lethal, as desired and as modeled in Figure 1.

### Effect of incomplete penetrance of lethality

**Figure 3:**
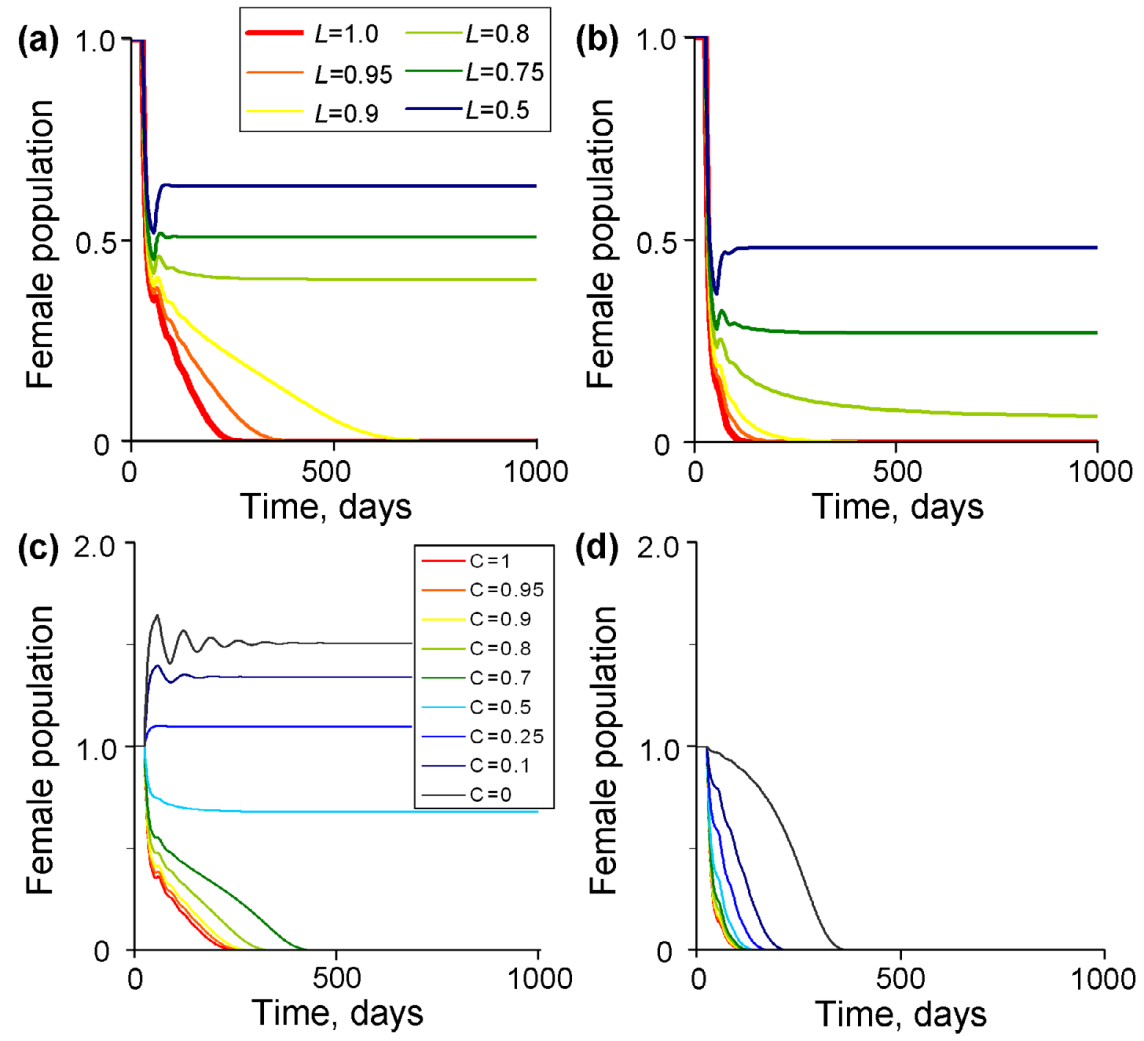
**Sensitivity to incomplete lethality or competitiveness**. We examined the impact of varying levels of lethality (penetrance) of the late-acting lethal (RIDL) system (a, b) or reduced competitiveness of the RIDL larvae (c, d) at release ratios of 2 (panel a) or 7.5 (panel b) with *β *= 1 and *P *= 1.31, equivalent to Fig. 1a and b respectively. Apart from the new parameters *L *and *C *(below), all other parameter values and assumptions about density-dependent mortality, relative mating competitiveness and release ratios are as for Fig. 1. (a, b) In each case, the lethality, *L*, associated with inheritance of a single copy of the RIDL construct was examined at values of *L *= 1, 0.95, 0.9, 0.8, 0.7 and 0.5. At *L *= 1, the outcome is identical to that of shown in Fig. 1 (red lines in Fig 1a, b). (c, d) The contribution of larvae carrying RIDL constructs to the overall density-dependent mortality experienced by all larvae in the generation was examined by varying a competitiveness scaling factor, *C*, between 1 (i.e. RIDL larvae are as competitive as the wild type and contribute equally to density-dependent mortality) and 0 (i.e. RIDL larvae contribute nothing to density-dependent mortality – this scenario is equivalent to an early acting conventional SIT system). *C *= 1 and *C *= 0 therefore correspond to the red and blue lines, respectively, in Fig. 1a,b.

Our model assumed 100% penetrant lethality; LA513A gave only 95–97%. We therefore extended the model to explore the effect of incomplete penetrance. As shown in Figure 3a–b, the effectiveness of a RIDL strategy using a late-acting lethal is not particularly sensitive to incomplete penetrance of the lethal phenotype even under the Dye's highest estimates of density dependent and growth rate parameters conditions (i.e. *β *= 1 and *P *= 1.31) [25]. In particular, leakiness at the 3–5% level shown for LA513A does not compromise the use or benefit of this strategy, relative to a hypothetical, fully penetrant strain (compare Fig 3a–b with Fig 1a–b; note change of axis scale). This is consistent with a study by Barclay [30] on the effect of incomplete sterility in a conventional SIT program, in which he concluded that moderate levels of non-sterility, e.g. 8%, would have little adverse effect, and that incomplete sterility was likely to be less of a problem for an SIT program directed against a population limited by density-dependent effects than if the target population were not so limited.

### Effect of imperfect competitiveness of "doomed" larvae

Another assumption of our model is that the "doomed" heterozygous larvae are fully competitive with wild type larvae. Mosquito larvae do not directly fight each other for food, rather they filter tiny particles of food from the surrounding water, so this seems a reasonable assumption. However, unless the transgene system is completely stage-specific, transgenic individuals may be weakened by the effect of the system before they are killed, and then might not be fully competitive with wild type during the density-dependent mortality phase. LA513A heterozygotes shows some lethality at the L4 stage, which may indicate this, though a study of density-dependent mortality in *Aedes aegypti *[31] suggested that most such mortality is at earlier larval stages. Any such lack of competitiveness would tend to reduce the benefit of late-acting lethality. This effect is explored in Fig. 3c–d. At the upper limit, *C *= 1, RIDL larvae are fully competitive, and the outcome is therefore identical to that of a late-acting lethal (red lines in Fig. 1a–b). At the lower limit, *C *= 0, RIDL larvae are completely uncompetitive, even if alive they contribute nothing to density-dependent mortality and so have no effect on the wild type larvae; the outcome is therefore identical to that of early-acting lethality (blue lines in Fig. 1a–b). Fig. 3c–d illustrates the effect of intermediate values of *C*. Most of the benefit of late-acting lethality is obtained at values of *C *= 0.7, in other words the effectiveness of the proposed strategy is not particularly sensitive to imperfect larval competitiveness.

## Conclusion

We have investigated, by mathematical modeling, the effect of adjusting the lethal phase in an SIT-like mosquito control program. We found that, for a population limited by density-dependent effects, delaying the time of death until after the density-dependent mortality phase would have a beneficial effect on program effectiveness, irrespective of assumptions about mass-release ratios or density-dependence parameters, though the effect is stronger for stronger density-dependence (Fig. 1). Our model does not associate any differential performance penalty with use of radiation or genetic engineering; the differential performance is solely due to adjusting the time of death. However, eliminating radiation may provide additional improvements. However, though radiation is generally highly damaging for mosquitoes [18,32,33], there may be exceptions [19] and the magnitude of the fitness penalty associated with genetic engineering, if any [34,35], is also somewhat controversial [34-38]. For density-dependent parameters estimated from field data for *Aedes aegypti*, the critical release ratio for population elimination is 27% to 540% greater for early-acting rather than late-acting lethality (540%, 210%, 270% and 27%, respectively, for the scenarios of Fig. 1c–f). This conclusion is also relatively insensitive to incomplete larval competitiveness or incomplete mortality, two likely imperfections in any real-world implementation of our proposed strategy (Fig. 3).

LA513A has a highly penetrant, fully repressible, late-acting, dominant lethal genetic system. This shows that it is possible to construct such a strain of a major disease vector, using current technology, and thereby makes the modeling and theoretical discussion relevant to real control programs and strategies. LA513B and LA882B, and most insertion lines of equivalent constructs in Medfly (*Ceratitis capitata*) and *Drosophila melanogaster *[[28] and data not shown] also show highly penetrant, fully repressible, dominant lethality. This indicates that this is a reproducible property of these constructs, and not particularly dependent on the precise insertion site (but not a universal property – LA882A showed no significant lethality even in the absence of tetracycline [data not shown]). However, time of death appears to be more sensitive to position effect in both *Aedes aegypti *and *Ceratitis capitata*, so a large panel of insertion lines might be required to find one that combines late-acting lethality with the other necessary characteristics for field use such as good flight ability, mating competitiveness, longevity in the field, etc. LA513A demonstrates the principle that transgenic strains may be constructed with repressible, dominant, late-acting lethality, but not that this is the only or optimal molecular strategy for achieving this phenotype. Rather than the "positive feedback" system of LA513, use of a late-acting promoter in a more conventional bipartite expression system might perhaps give more reliable control over the time of expression of an effector molecule and hence the lethal phase; this has previously been demonstrated in *Drosophila *for female-specific promoters and for embryo-specific promoters [27,39,40].

The recent development of genetic transformation methods for mosquito species has opened the door to a range of approaches to the control of disease transmission that are based on manipulating the genome of the mosquito vector [41]. Population replacement strategies, which aim to convert a pathogen-transmitting mosquito population into one incapable of transmission, may provide the best solution in the long term, particularly for poorer regions with very large mosquito populations. However, genetics-based population suppression and elimination strategies, such as RIDL and conventional SIT, have considerable potential applicability in many parts of the world. Since it requires rather simpler molecular biology and genetics, RIDL may be available much earlier than systems based on population replacement. From a regulatory point of view RIDL is also somewhat less challenging, as an autocidal system will rapidly eliminate itself from the environment unless deliberately maintained by constant re-introduction. It has therefore been suggested that SIT should be the first application for field release of transgenic insects [10]. We have shown here that a mosquito strain with the genetic properties (late-acting, repressible dominant lethality) necessary for the RIDL strategy we propose can indeed be constructed, and that, for mosquitoes, this system has significant theoretical advantages over classical SIT. However, political action has derailed at least one mosquito SIT program in the past [42,43], and it will be essential to obtain broad political support and regulatory approval if this method is indeed to help to reduce the burden of vector-borne disease.

## Materials and methods

### Mathematical model of mosquito population dynamics

The dynamics of the wild type adult mosquito population, *A*, through time, *t*, are described by a time-delayed differential equation model (which captures the overlapping generations characteristic of *Ae. aegypti*), with a two-parameter, *α *and *β*, density-dependent function [25]. The model, which assumes density-dependent mortality acts on pre-adultlife stages, takes the form

dAtdt=PAt−Texp⁡[−α(At−TE)β]−δAt     (Eq. 1)
 MathType@MTEF@5@5@+=feaafiart1ev1aaatCvAUfKttLearuWrP9MDH5MBPbIqV92AaeXatLxBI9gBaebbnrfifHhDYfgasaacH8akY=wiFfYdH8Gipec8Eeeu0xXdbba9frFj0=OqFfea0dXdd9vqai=hGuQ8kuc9pgc9s8qqaq=dirpe0xb9q8qiLsFr0=vr0=vr0dc8meaabaqaciaacaGaaeqabaqabeGadaaakeaadaWcaaqaaiabbsgaKjabdgeabnaaBaaaleaacqWG0baDaeqaaaGcbaGaeeizaqMaemiDaqhaaiabg2da9iabdcfaqjabdgeabnaaBaaaleaacqWG0baDcqGHsislcqWGubavaeqaaOGagiyzauMaeiiEaGNaeiiCaa3aamWaaeaacqGHsisliiGacqWFXoqydaqadaqaaiabdgeabnaaBaaaleaacqWG0baDcqGHsislcqWGubavaeqaaOGaemyraueacaGLOaGaayzkaaWaaWbaaSqabeaacqWFYoGyaaaakiaawUfacaGLDbaacqGHsislcqWF0oazcqWGbbqqdaWgaaWcbaGaemiDaqhabeaakiaaxMaacaWLjaWaaeWaaeaacqqGfbqrcqqGXbqCcqqGUaGlcqqGGaaicqaIXaqmaiaawIcacaGLPaaaaaa@5994@

in which *T *is the mosquito generation time, *δ *is the per capita daily adult death rate, *E *is the maximum per capita daily egg production rate, and *P *is the maximum per capita daily egg production rate corrected for density-independent egg to adult survival. We assume a 1:1 sex ratio.

In the absence of any control, the maximum finite rate of increase (net fecundity after lifetime density-independent mortalities), *λ*, is

λ=Pδ     (Eq. 2)
 MathType@MTEF@5@5@+=feaafiart1ev1aaatCvAUfKttLearuWrP9MDH5MBPbIqV92AaeXatLxBI9gBaebbnrfifHhDYfgasaacH8akY=wiFfYdH8Gipec8Eeeu0xXdbba9frFj0=OqFfea0dXdd9vqai=hGuQ8kuc9pgc9s8qqaq=dirpe0xb9q8qiLsFr0=vr0=vr0dc8meaabaqaciaacaGaaeqabaqabeGadaaakeaaiiGacqWF7oaBcqGH9aqpdaWcaaqaaiabdcfaqbqaaiab=r7aKbaacaWLjaGaaCzcamaabmaabaGaeeyrauKaeeyCaeNaeeOla4IaeeiiaaIaeGOmaidacaGLOaGaayzkaaaaaa@3A29@

and the equilibrium adult population size, A*, is

A∗=[ln⁡(P/δ)α]1βE     (Eq. 3)
 MathType@MTEF@5@5@+=feaafiart1ev1aaatCvAUfKttLearuWrP9MDH5MBPbIqV92AaeXatLxBI9gBaebbnrfifHhDYfgasaacH8akY=wiFfYdH8Gipec8Eeeu0xXdbba9frFj0=OqFfea0dXdd9vqai=hGuQ8kuc9pgc9s8qqaq=dirpe0xb9q8qiLsFr0=vr0=vr0dc8meaabaqaciaacaGaaeqabaqabeGadaaakeaacqWGbbqqdaahaaWcbeqaaiabgEHiQaaakiabg2da9maalaaabaWaamWaaeaadaWcaaqaaiGbcYgaSjabc6gaUnaabmaabaGaemiuaaLaei4la8ccciGae8hTdqgacaGLOaGaayzkaaaabaGae8xSdegaaaGaay5waiaaw2faamaaCaaaleqabaWaaSaaaeaacqaIXaqmaeaacqWFYoGyaaaaaaGcbaGaemyraueaaiaaxMaacaWLjaWaaeWaaeaacqqGfbqrcqqGXbqCcqqGUaGlcqqGGaaicqaIZaWmaiaawIcacaGLPaaaaaa@4764@

In evaluating the performance of the conventional SIT (early-acting lethality) and RIDL (late-acting lethality) systems we assume the population is at pre-control stable equilibrium when SIT control is initiated (i.e. *A*_0 _= A*. The input ratio, *I*, of released males is defined relative to the pre-control population equilibrium, A*, such that the number of "sterile" males remains constant through time.

Under both release scenarios, females are assumed to select mates proportionately to their relative abundance. Therefore, for the constant number release scenario the proportion of females that mate with wild type males at time *t *is *A*_*t*_/(*A*_*t *_+ IA*).

With the early-acting lethal, we assume that the all offspring of sterile males die at early embryogenesis and so do not contribute to density-dependent mortality in pre-adult life stages. The dynamics of the mosquito population under conventional SIT control is given by

dAtdt=(PAt−TAt−TAt−T+IA∗)exp⁡[−α(At−TAt−TAt−T+IA∗E)β]−δAt.     (Eq. 4)
 MathType@MTEF@5@5@+=feaafiart1ev1aaatCvAUfKttLearuWrP9MDH5MBPbIqV92AaeXatLxBI9gBaebbnrfifHhDYfgasaacH8akY=wiFfYdH8Gipec8Eeeu0xXdbba9frFj0=OqFfea0dXdd9vqai=hGuQ8kuc9pgc9s8qqaq=dirpe0xb9q8qiLsFr0=vr0=vr0dc8meaabaqaciaacaGaaeqabaqabeGadaaakeaadaWcaaqaaiabbsgaKjabdgeabnaaBaaaleaacqWG0baDaeqaaaGcbaGaeeizaqMaemiDaqhaaiabg2da9maabmaabaGaemiuaaLaemyqae0aaSbaaSqaaiabdsha0jabgkHiTiabdsfaubqabaGcdaWcaaqaaiabdgeabnaaBaaaleaacqWG0baDcqGHsislcqWGubavaeqaaaGcbaGaemyqae0aaSbaaSqaaiabdsha0jabgkHiTiabdsfaubqabaGccqGHRaWkcqWGjbqscqWGbbqqdaahaaWcbeqaaiabgEHiQaaaaaaakiaawIcacaGLPaaacyGGLbqzcqGG4baEcqGGWbaCdaWadaqaaiabgkHiTGGaciab=f7aHnaabmaabaGaemyqae0aaSbaaSqaaiabdsha0jabgkHiTiabdsfaubqabaGcdaWcaaqaaiabdgeabnaaBaaaleaacqWG0baDcqGHsislcqWGubavaeqaaaGcbaGaemyqae0aaSbaaSqaaiabdsha0jabgkHiTiabdsfaubqabaGccqGHRaWkcqWGjbqscqWGbbqqdaahaaWcbeqaaiabgEHiQaaaaaGccqWGfbqraiaawIcacaGLPaaadaahaaWcbeqaaiab=j7aIbaaaOGaay5waiaaw2faaiabgkHiTiab=r7aKjabdgeabnaaBaaaleaacqWG0baDaeqaaOGaeiOla4IaaCzcaiaaxMaadaqadaqaaiabbweafjabbghaXjabb6caUiabbccaGiabisda0aGaayjkaiaawMcaaaaa@77C3@

In contrast, for late acting lethality, we assume all offspring of "sterile" males survive through the pre-adult life stages and contribute fully to density-dependent mortality, before dying prior to adult emergence. The resulting population dynamics under RIDL control are described by

dAt−Tdt=(PAt−TAt−TAt−T+IA∗)exp⁡[−α(At−TE)β]−δAt.     (Eq. 5)
 MathType@MTEF@5@5@+=feaafiart1ev1aaatCvAUfKttLearuWrP9MDH5MBPbIqV92AaeXatLxBI9gBaebbnrfifHhDYfgasaacH8akY=wiFfYdH8Gipec8Eeeu0xXdbba9frFj0=OqFfea0dXdd9vqai=hGuQ8kuc9pgc9s8qqaq=dirpe0xb9q8qiLsFr0=vr0=vr0dc8meaabaqaciaacaGaaeqabaqabeGadaaakeaadaWcaaqaaiabbsgaKjabdgeabnaaBaaaleaacqWG0baDcqGHsislcqWGubavaeqaaaGcbaGaeeizaqMaemiDaqhaaiabg2da9maabmaabaGaemiuaaLaemyqae0aaSbaaSqaaiabdsha0jabgkHiTiabdsfaubqabaGcdaWcaaqaaiabdgeabnaaBaaaleaacqWG0baDcqGHsislcqWGubavaeqaaaGcbaGaemyqae0aaSbaaSqaaiabdsha0jabgkHiTiabdsfaubqabaGccqGHRaWkcqWGjbqscqWGbbqqdaahaaWcbeqaaiabgEHiQaaaaaaakiaawIcacaGLPaaacyGGLbqzcqGG4baEcqGGWbaCdaWadaqaaiabgkHiTGGaciab=f7aHnaabmaabaGaemyqae0aaSbaaSqaaiabdsha0jabgkHiTiabdsfaubqabaGccqWGfbqraiaawIcacaGLPaaadaahaaWcbeqaaiab=j7aIbaaaOGaay5waiaaw2faaiabgkHiTiab=r7aKjabdgeabnaaBaaaleaacqWG0baDaeqaaOGaeiOla4IaaCzcaiaaxMaadaqadaqaaiabbweafjabbghaXjabb6caUiabbccaGiabiwda1aGaayjkaiaawMcaaaaa@6C05@

For any value of *I *> 0, the population will move away from A* towards a new, control-mediated equilibrium. If the release ratio exceeds a critical threshold, I*, the wild type population will be driven to extinction (i.e. control-mediated equilibrium = 0).

The relative performance of the conventional SIT and RIDL systems was examined by simulating equations 4 and 5 over a range of values of *I *and for different combinations of parameters values for *β *(0.302 and 1) and *P *(0.367 and 1.31), representing the range estimated by Dye [25]. For each combination of *β *and *P*, the critical release ratio for population eradication was estimated numerically for both early and late lethality approaches. The simulation was conducted using a one-day time step; at each time step the magnitude of *A*_*t *_was divided by A* so as to scale the wild type adult population relative to the pre-control equilibrium. This relative measure of adult population numbers is independent of the magnitude of *α *and *E *(for all non-zero values of *α *and *E*) and is equivalent to the relative adult female mosquito population (as the egg sex ratio is equal). A simulation approach was adopted because there are no analytical solutions for the critical release ratios, nor the equilibrium population sizes for values of *I *> 0.

To model the effect of incomplete penetrance of RIDL-induced mortality, the equation describing the dynamics of mosquito population under RIDL control (equation 5) was adjusted to account for the proportion of lethality, *L*, giving

dAtdt=[PAt−TAt−TAt−T+IA*+PAt−TIA∗At−T+IA∗(1−L)]exp⁡[−α(At−TE)β]−δAt.     (Eq. 6)
 MathType@MTEF@5@5@+=feaafiart1ev1aaatCvAUfKttLearuWrP9MDH5MBPbIqV92AaeXatLxBI9gBaebbnrfifHhDYfgasaacH8akY=wiFfYdH8Gipec8Eeeu0xXdbba9frFj0=OqFfea0dXdd9vqai=hGuQ8kuc9pgc9s8qqaq=dirpe0xb9q8qiLsFr0=vr0=vr0dc8meaabaqaciaacaGaaeqabaqabeGadaaakeaadaWcaaqaaiabbsgaKjabdgeabnaaBaaaleaacqWG0baDaeqaaaGcbaGaeeizaqMaemiDaqhaaiabg2da9maadmaabaGaemiuaaLaemyqae0aaSbaaSqaaiabdsha0jabgkHiTiabdsfaubqabaGcdaWcaaqaaiabdgeabnaaBaaaleaacqWG0baDcqGHsislcqWGubavaeqaaaGcbaGaemyqae0aaSbaaSqaaiabdsha0jabgkHiTiabdsfaubqabaGccqGHRaWkcqWGjbqscqWGbbqqdaahaaWcbeqaaiabcQcaQaaaaaGccqGHRaWkcqWGqbaucqWGbbqqdaWgaaWcbaGaemiDaqNaeyOeI0IaemivaqfabeaakmaalaaabaGaemysaKKaemyqae0aaWbaaSqabeaacqGHxiIkaaaakeaacqWGbbqqdaWgaaWcbaGaemiDaqNaeyOeI0IaemivaqfabeaakiabgUcaRiabdMeajjabdgeabnaaCaaaleqabaGaey4fIOcaaaaakmaabmaabaGaeGymaeJaeyOeI0IaemitaWeacaGLOaGaayzkaaaacaGLBbGaayzxaaGagiyzauMaeiiEaGNaeiiCaa3aamWaaeaacqGHsisliiGacqWFXoqydaqadaqaaiabdgeabnaaBaaaleaacqWG0baDcqGHsislcqWGubavaeqaaOGaemyraueacaGLOaGaayzkaaWaaWbaaSqabeaacqWFYoGyaaaakiaawUfacaGLDbaacqGHsislcqWF0oazcqWGbbqqdaWgaaWcbaGaemiDaqhabeaakiabc6caUiaaxMaacaWLjaWaaeWaaeaacqqGfbqrcqqGXbqCcqqGUaGlcqqGGaaicqaI2aGnaiaawIcacaGLPaaaaaa@81FB@

To model the effect of reduced competitiveness of RIDL larvae, relative to wild type, equation 5 was adjusted to incorporate a competitiveness scaling factor, *C*, which can take values between 1 (RIDL larvae are fully competitive and contribute equally to density-dependent mortality) and 0 (RIDL larvae contribute nothing to density-dependent mortality giving

dAtdt=[PAt−TAt−TAt−T+IA∗]exp⁡[−α(At−T(At−TAt−T+IA∗+IA∗At−T+IA∗C)E)β]−δAt     (Eq. 7)
 MathType@MTEF@5@5@+=feaafiart1ev1aaatCvAUfKttLearuWrP9MDH5MBPbIqV92AaeXatLxBI9gBaebbnrfifHhDYfgasaacH8akY=wiFfYdH8Gipec8Eeeu0xXdbba9frFj0=OqFfea0dXdd9vqai=hGuQ8kuc9pgc9s8qqaq=dirpe0xb9q8qiLsFr0=vr0=vr0dc8meaabaqaciaacaGaaeqabaqabeGadaaakeaadaWcaaqaaiabbsgaKjabdgeabnaaBaaaleaacqWG0baDaeqaaaGcbaGaeeizaqMaemiDaqhaaiabg2da9maadmaabaGaemiuaaLaemyqae0aaSbaaSqaaiabdsha0jabgkHiTiabdsfaubqabaGcdaWcaaqaaiabdgeabnaaBaaaleaacqWG0baDcqGHsislcqWGubavaeqaaaGcbaGaemyqae0aaSbaaSqaaiabdsha0jabgkHiTiabdsfaubqabaGccqGHRaWkcqWGjbqscqWGbbqqdaahaaWcbeqaaiabgEHiQaaaaaaakiaawUfacaGLDbaacyGGLbqzcqGG4baEcqGGWbaCdaWadaqaaiabgkHiTGGaciab=f7aHnaabmaabaGaemyqae0aaSbaaSqaaiabdsha0jabgkHiTiabdsfaubqabaGcdaqadaqaamaalaaabaGaemyqae0aaSbaaSqaaiabdsha0jabgkHiTiabdsfaubqabaaakeaacqWGbbqqdaWgaaWcbaGaemiDaqNaeyOeI0IaemivaqfabeaakiabgUcaRiabdMeajjabdgeabnaaCaaaleqabaGaey4fIOcaaaaakiabgUcaRmaalaaabaGaemysaKKaemyqae0aaWbaaSqabeaacqGHxiIkaaaakeaacqWGbbqqdaWgaaWcbaGaemiDaqNaeyOeI0IaemivaqfabeaakiabgUcaRiabdMeajjabdgeabnaaCaaaleqabaGaey4fIOcaaaaakiabdoeadbGaayjkaiaawMcaaiabdweafbGaayjkaiaawMcaamaaCaaaleqabaGae8NSdigaaaGccaGLBbGaayzxaaGaeyOeI0Iae8hTdqMaemyqae0aaSbaaSqaaiabdsha0bqabaGccaWLjaGaaCzcamaabmaabaGaeeyrauKaeeyCaeNaeeOla4IaeeiiaaIaeG4naCdacaGLOaGaayzkaaaaaa@8722@

*C *can take values between 1 (RIDL larvae are fully competitive and contribute equally to density-dependent mortality) and 0 (RIDL larvae contribute nothing to density-dependent mortality).

### Mosquito transformation and rearing

*Aedes aegypti *of the Rockefeller strain (obtained from Roger Wood, University of Manchester), were reared in an insectary maintained at 28°C and 75–80% relative humidity with 12-hour light/dark cycle. Mosquitoes were transformed by standard micro-injection methods [44], using a vector plasmid (e.g. pLA513) concentration of 500 ng/μl coinjected with a 400 ng/μl concentration of *piggyBac *'helper plasmid' phsp-pBac [45] as the source of *piggyBac *transposase. After injection, eggs were heat shocked at 37°C for 2 hours, then stored for 48 hours at 100% humidity before they were submerged for hatching. Adult injection survivors ('Generation 0' or G_0_) were back-crossed to wild type: individual G_0 _males were crossed to 10–15 wild type females and G_0 _females were combined in pools of 10 with 3 wild type males. G_1 _eggs were collected and hatched as above (but without heat-shock). Hatched G_1 _larvae were screened for fluorescence using an Olympus SZX-12 microscope equipped for fluorescence (filters for red fluorescence: excitation 510–550, emission 590LP). Two independent transgenic lines, designated LA513A and LA513B, were recovered from about 200 fertile G_0 _back crosses, representing a transformation efficiency of approximately 1%. This is lower than published *piggyBac*-mediated transformation rates for *Aedes aegypti *of 8–11% [46,47]. This decrease may reflect the larger size of the LA513 construct, some loss of some transgenics due to the deleterious effect of overexpression of tTAV, and/or variations in experimental technique or environment. DsRed2 fluorescence could be observed in the thorax of all developmental stages of LA513A mosquitoes. For the experiments in Table 1, larvae were reared at 200–250 larvae per liter, with 5–8 pellets of fish food (Omega) every 2 days. Tetracycline (Sigma) was added to the larval water to a final concentration of 30 μg/ml, as appropriate. Eggs were washed carefully after collection to minimize carry-over of tetracycline from one generation to the next. Males and females were separated as pupae to ensure female virginity for all experimental crosses.

### Molecular analysis

Inverse PCR to identify genomic sequence adjacent to the insertion site of LA513A was performed as Handler et al. [45], using HaeIII, MspI and TaqI restriction enzymes. Inverse PCR products were cloned into pCRII-TOPO (Invitrogen, Paisley, UK) prior to sequencing.

## List of abbreviations used

SIT: Sterile Insect Technique

RIDL: Release of Insects carrying a Dominant Lethal

## Figures and Tables

**Table 1:** Tetracycline-repressible lethality in LA513A.

Cross	Parents		Progeny									
	Male	Female	Tc	Genotype	Egg	L1	L2	L3	L4	Pupae	Adults	%

a	LA513A/+	+/+	Yes	LA513A/+	1000	489	468	446	442	437	434	89
				Wild type		444	431	403	400	396	392	88
b	+/+	LA513A/+	Yes	LA513A/+	1000	442	420	404	399	393	383	87
				Wild type		466	444	428	417	412	404	87
c	LA513A/+	+/+	No	LA513A/+	540	274	265	235	208	155	**7**	**2.6**
				Wild type		233	225	214	212	209	206	88
d	+/+	LA513A/+	No	LA513A/+	497	216	205	181	168	131	**9**	**4.2**
				Wild type		241	225	216	214	211	207	86

Adults heterozygous for LA513A were allowed to mate with wild type. a and c: transgenic males crossed to wild type females; b, d: the reciprocal cross using transgenic females. Eggs were collected and the resulting larvae reared in media supplemented with tetracycline (Tc) to 30 μg/ml (crosses a and b) or in normal, tetracycline-free media (crosses c and d). Data are the sum of at least 5 experiments. The ratio of transgenic to non-transgenic first instar larvae (L1) was approximately 1:1 (1421:1384), indicating that there was no significant differential embryonic mortality between transgenic and wild type (binomial exact test,*p *= 0.248). The numbers of transgenic and non-transgenic first, second, third and fourth instar larvae (L1–L4), pupae and adults resulting from these eggs is shown. In the absence of tetracycline, the transgenics showed very high (96–97%) mortality between first instar larvae and adult stages (highlighted cells); this was completely suppressed by tetracycline. Mortality of transgenics in the absence of tetracycline is strongly stage-specific, being primarily around the pupal stage (L4-pupae and pupae-adult; most affected individuals started to pupate but failed to develop beyond the earliest stages of pupal development).
